# Differential Effects of Itaconate and its Esters on the Glutathione and Glucose Metabolism of Cultured Primary Rat Astrocytes

**DOI:** 10.1007/s11064-024-04263-0

**Published:** 2024-11-20

**Authors:** Patrick Watermann, Gurleen K. Kalsi, Ralf Dringen, Christian Arend

**Affiliations:** 1 Centre for Biomolecular Interactions Bremen, Faculty 2 (Biology/Chemistry), University of Bremen, 28359 Bremen, Germany; 2 Centre for Environmental Research and Sustainable Technology, University of Bremen, 28359 Bremen, Germany

**Keywords:** Astrocytes, Glutathione, Glycolysis, Itaconate, Pentose-phosphate pathway

## Abstract

Itaconate is produced as endogenous metabolite by decarboxylation of the citric acid cycle intermediate *cis*-aconitate. As itaconate has anti-microbial and anti-inflammatory properties, this substance is considered as potential therapeutic drug for the treatment of inflammation in various diseases including traumatic brain injury and stroke. To test for potential adverse effects of itaconate on the viability and metabolism of brain cells, we investigated whether itaconate or its membrane permeable derivatives dimethyl itaconate (DI) and 4-octyl itaconate (OI) may affect the basal glucose and glutathione (GSH) metabolism of cultured primary astrocytes. Acute exposure of astrocytes to itaconate, DI or OI in concentrations of up to 300 µM for up to 6 h did not compromise cell viability. Of the tested substances, only OI stimulated aerobic glycolysis as shown by a time- and concentration-dependent increase in glucose-consumption and lactate release. None of the tested itaconates affected the pentose-phosphate pathway-dependent reduction of the water-soluble tetrazolium salt 1 (WST1). In contrast, both DI and OI, but not itaconate, depleted cellular GSH in a time- and concentration-dependent manner. For OI this depletion was accompanied by a matching increase in the extracellular GSH content that was completely prevented in the presence of the multidrug resistance protein 1 (Mrp1)-inhibitor MK571, while in DI-treated cultures GSH was depleted both in cells and medium. These data suggest that OI stimulates Mrp1-mediated astrocytic GSH export, while DI reacts with GSH to a conjugate that is not detectable by the GSH assay applied. The data presented demonstrate that itaconate, DI and OI differ strongly in their effects on the GSH and glucose metabolism of cultured astrocytes. Such results should be considered in the context of the discussed potential use of such compounds as therapeutic agents.

## Introduction

Itaconic acid (2-methylidenebutanedioic acid) is a mammalian metabolite that is produced in stimulated macrophages [1]. The enzyme *cis*-aconitate decarboxylase generates itaconate by decarboxylation of the citric acid cycle intermediate *cis*-aconitate, thereby linking in macrophages mitochondrial metabolism to immunity [2]. Itaconate is a dicarboxylic acid that contains five carbon atoms and an α,β-unsaturated alkene structure. Due to its two negatively charged carboxylate groups, itaconate applied to cells is considered to possess low membrane permeability. To overcome this problem, itaconate esters with higher lipophilicity and membrane permeability such as dimethyl itaconate (DI, dimethyl 2-methylidenebutanedioate) or 4-octyl itaconate (OI, 2-methylidene-4-octoxy-4-oxobutanoic acid) are frequently used to study pharmacological effects of itaconate in vivo and in vitro [3]. The finding that itaconate can activate the Nrf2 signaling pathways by alkylation of Keap1, thereby initiating the transcription of pro-inflammatory and anti-oxidant genes, has made itaconate a promising candidate for therapeutic applications in a variety of diseases and tissue types [4]. In the context of the brain, itaconate is currently considered for treatments of traumatic brain injury, stroke and neuroinflammation-associated depression [4, 5]. Moreover, itaconate has very recently been shown to be secreted from activated macrophages and activates an innate immune response signaling pathway in the respiratory epithelium by activation of oxoglutarate receptor 1 (OXGR1) [6].

The brain consists mainly of neurons and glial cells, whereby astrocytes represent the majority of glial cells [7]. These cells hold a pivotal role in the brain’s normal and drug-related metabolism [8–10]. As astrocytes are deeply involved and differentially activated in several neurological disorders including ischemic stroke, traumatic brain injury and Alzheimer’s disease, they are considered as promising therapeutic target [11–15]. Moreover, astrocytic end-feet cover the brain capillaries nearly completely and, as a consequence, astrocytes are the first parenchymal brain cell type that encounters substances delivered by the bloodstream and cross the blood-brain barrier [16]. Importantly, astrocytes are also tightly associated with neuronal synapses [17, 18] and take up extracellular neurotransmitters from the synaptic cleft thereby maintaining synaptic connectivity [17, 18]. Furthermore, astrocytes supply neurons with precursors for glutathione (GSH) synthesis [19, 20].

The tripeptide GSH (γ-glutamyl-cysteinyl-glycine) is an important antioxidant that is present in the cytosol of cultured astrocytes in a concentration of 8 mM [21]. GSH is oxidized to glutathione disulfide (GSSG) during oxidative stress [22, 23]. This oxidation provides electrons that are used for the reduction of reactive oxygen species (ROS) [24, 25]. The accumulating GSSG is rapidly reduced to GSH by the NADPH-dependent glutathione reductase [26, 27] or, if GSSG reduction is limited, can be exported from cultured astrocytes to slow-down the rapid oxidation of the cellular thiol reduction potential [24, 25]. Astrocytes do also export GSH as first step of the supply of precursor molecules for GSH synthesis in neighbouring neurons [19, 20, 25]. Furthermore, GSH is a key player in the cellular detoxification of organic compounds, since it can be conjugated to various types of xenobiotics via glutathione-*S*-transferases. This helps to detoxify potentially harmful substances, which are subsequently exported from the cells via multidrug-resistance proteins [25, 28, 29]. The multidrug-resistance protein 1 (Mrp1) is, together with other ABC-family members, expressed in primary astrocyte cultures [22, 30] and has been demonstrated to mediate the export of GSH [30, 31] and GSSG from these cells [22, 23, 30, 31]. The export of these two Mrp1 substrates from astrocyte cultures has been reported to be efficiently inhibited by the Mrp1 inhibitor MK571 [22, 23, 30, 32–34].

So far little information is available on the consequences of an exposure of astrocytes to itaconate. Application of itaconate in a high concentration of 2 mM promotes succinate accumulation by reversible inhibition of succinate dehydrogenase activity in vitro in primary astrocyte cultures [35]. For these conditions, an increase in the intracellular amounts of itaconate to around 3 nmol/well was detected, suggesting that itaconate is able to enter astrocytes, at least if applied in millimolar concentrations [35]. Very recently, OI was reported to enhance the expression of Nrf2 and its nuclear translocation in astrocytes in an in vivo mouse hypoxic ischemic model [36]. Also after incubation of primary astrocytes with DI, activation of Nrf2 was reported in addition to increased activities of the antioxidative enzymes catalase and superoxide dismutase and an elevated GSH/GSSG ratio [37].

In order to investigate whether an acute exposure with itaconate or its derivatives DI or OI may affect the GSH or glucose metabolism of brain cells, cultured primary astrocyte cultures were incubated in the absence or presence of either of these three compounds and the cellular and extracellular amounts of GSH and GSSG, the glucose consumption, the lactate release and the ability to provide electrons via the pentose phosphate pathway (PPP) for WST1 reduction were investigated. Here we report that under the conditions applied, the presence of itaconate had no modulatory effect on the investigated metabolic parameters. In contrast, DI strongly reduced the amounts of detectable cellular and extracellular GSH, most likely by conjugation via a Michael-type reaction, while OI stimulated the Mrp1-mediated export of GSH and the glycolytic lactate production.

## Experimental Procedures

### Materials and Methods

Itaconic acid (I29204), dimethyl itaconate (DI; 592498) and fetal calf serum (F7524) were purchased from Sigma-Aldrich (Steinheim, Germany, RRID: SCR_008988). 4-Octyl itaconate (OI; 25374) and G6PDi-1 (31484) were from Cayman Chemical (Tallinn, Estonia; RRID: SCR_008945). MK-571 (MK571; AG-CR1-0021) was purchased form AdipoGen Life Sciences (San Diego, USA; RRID: SCR_010507). WST1 (W201) was from Dojindo (Munich, Germany) and β-lapachone (ab141097) from Abcam (Berlin, Germany; RRID: SCR_012931). Acivicin (sc-200498) was bought from Santa Cruz Biotechnology (Heidelberg, Germany; RRID: SCR_008987). Dulbecco’s modified Eagle’s medium (DMEM containing 25 mM glucose; 12100061) and penicillin G/streptomycin sulfate solution (15140122) was obtained from Thermo Fisher Scientific (Schwerte, Germany; RRID: SCR_008452). Sulfosalicylic acid (SSA; A0416), bovine serum albumin (BSA; A1391), NADPH (A1395) and NADH (A1393) were purchased from Applichem (Darmstadt, Germany; RRID: SRC_005814). The enzyme glutathione reductase (G3664) was from Sigma-Aldrich (Steinheim, Germany, RRID: SCR_008988). The enzymes glucose-6-phosphate dehydrogenase (10165875001), hexokinase (11426362001), glutamate-pyruvate transaminase (10737127001) and lactate dehydrogenase (10127876001) were obtained from Roche Diagnostics (Mannheim, Germany; RRID: SCR_001326). All other chemicals of the highest purity available were obtained from Merck (Darmstadt, Germany; RRID: SCR_001287), Sigma-Aldrich (Steinheim, Germany; RRID: SCR_008988), Fluka (Buchs, Switzerland), Roth (Karlsruhe, Germany; RRID: SCR_005711) or Riedel-de-Haën (Seelze, Germany). Sterile cell culture plates and unsterile 96-well microtiter plates were from Sarstedt (Nümbrecht, Germany).

### Astrocyte Cultures

Brains of newborn Wistar rats were used to prepare astrocyte-rich primary cultures as previously described in detail [38]. Briefly, after mechanical dissociation of brain tissue, the suspension of harvested cells was diluted in culture medium (90% DMEM containing 25 mM glucose, 20 U/mL penicillin G, 20 µg/mL streptomycin, 44.6 mM NaHCO_3_ and 1 mM pyruvate supplemented with 10% fetal calf serum) and 300 000 viable cells in 1 mL medium were seeded into wells of 24-well plates. The cultures were incubated at 37 °C in a humidified atmosphere in a Sanyo incubator (Osaka, Japan) supplying 10% CO_2_. The culture medium was renewed every 7th day and 24 h prior to each experiment. All experiments for the presented study were performed on confluent cultures of an age between 17 and 27 days. Most cells in these cultures are astrocytes and only low amounts of contaminating oligodendrocytes and microglial cells are present [38, 39].

### Experimental Incubation of the Cells

The cells were washed twice with 1 mL prewarmed (37 °C) incubation buffer (IB; 20 mM HEPES, 5 mM D-glucose, 145 mM NaCl, 5.4 mM KCl, 1.8 mM CaCl_2_, 1 mM MgCl_2_, 0.8 mM Na_2_HPO_4_, pH adjusted with NaOH at 37 °C to 7.4) and incubated at 37 °C with 200 µL IB containing 100 µM of the γ-glutamyltranspeptidase inhibitor acivicin [40] in the absence or the presence of the compounds indicated in the figures and tables. After the given incubation periods, the media were collected for determination of extracellular levels of GSx, GSSG, glucose and lactate as well as extracellular LDH activity. The cells were washed with 1 mL of ice-cold phosphate-buffered saline (PBS; 10 mM potassium phosphate buffer, pH 7.4, containing 150 mM NaCl) before the extracellular and cellular contents of GSx (GSx = amount of GSH plus twice the amount of GSSG) and GSSG were determined.

### Cell-free Incubation of GSH with Itaconate and its Derivatives

GSH (10 µM) was incubated without (0 µM) or with 1 mM or 10 mM itaconate, DI or OI for up to 4 h in 1 mL IB in wells of a 24-well dish at room temperature. Afterwards, the GSH content was determined as described below.

### Determination of Cell Viability and Cellular Protein Content

Lactate dehydrogenase (LDH) is a cytosolic enzyme that is released from cells upon impairment of membrane integrity. Hence, extracellular LDH activity has been monitored as indication for a potential loss in cell viability as previously described in detail [38, 41]. To calculate specific metabolite values, the data for the contents of GSx and GSSG, lactate release, glucose consumption and WST1 formazan formation were normalized to the protein content of the individual cultures, which was measured by the Lowry method [42] using BSA as standard protein.

### Determination of the Contents of GSx and GSSG

The levels of cellular and extracellular GSx (amount of GSH + twice the amount of GSSG) and GSSG were assessed using a modified version of the colorimetric Tietze method adapted for 96-well microtiter plates, as previously described [38]. Cell lysis was performed using 200 µL of 1% (w/v) sulfosalicylic acid and 10 µL of these lysates were utilized to quantify the cellular GSx and GSSG levels. To determine the extracellular GSx or GSSG contents, 10 µL of media samples were combined with 10 µL of 1% (w/v) sulfosalicylic acid, and this mixture was applied to the assays. The contribution of GSSG to the GSx values determined was quantified after removal of GSH from the samples by derivatization to 2-vinylpyridine as previous described [38].

### WST1 Reduction by Astrocyte Cultures

To examine the impact of itaconate, DI or OI on cell-mediated WST1 reduction, astrocyte cultures were washed twice with 1 mL of pre-warmed (37 °C) glucose-free IB and then preincubated for 30 min at 37 °C with 300 µL of glucose-free IB. Following a subsequent washing step with 300 µL of glucose-free IB, the cells were exposed to 300 µL of IB containing no or 5 mM glucose, in the presence of 3 µM of the redox cycler β-lapachone, 400 µM WST1 and itaconate, DI or OI at the indicated concentrations for up to 60 min in a humidified atmosphere (without CO_2_) in a cell incubator. After the given incubation periods, the incubation media were collected, and 50 µL samples were diluted with water to a total volume of 200 µL in the wells of a microtiter plate. The absorbance of the WST1 formazan produced was measured at 450 nm using a microtiter plate reader (Multiscan Sky, Thermo Fisher, Darmstadt, Germany) as previously reported [43, 44]. The specific accumulation of WST1 formazan was determined by normalizing the extracellular WST1 formazan content to the initial protein content of the respective cultures.

### Glucose and Lactate Determination in Incubation Media

The glucose and lactate concentration in the incubation media were measured before and after the indicated periods of incubation using coupled enzymatic assays, as described previously [38]. Glucose utilization was determined by subtracting the initial glucose concentration in the incubation buffer from the concentration measured after the given incubation period.

### Presentation of Data

The presented data are means ± standard deviation (SD) of values derived from experiments conducted in independent experiments on three separately prepared astrocyte cultures. Statistical analysis for differences among multiple data groups was performed using ANOVA followed by the Bonferroni post-hoc test. The t-test was used for comparing two data sets. A p-value greater than 0.05 was considered not significant.

## Results

### Investigation of a Potential Alteration of the Astrocytic GSH Content and Export by Itaconate or its Derivatives

**Fig. 1:**
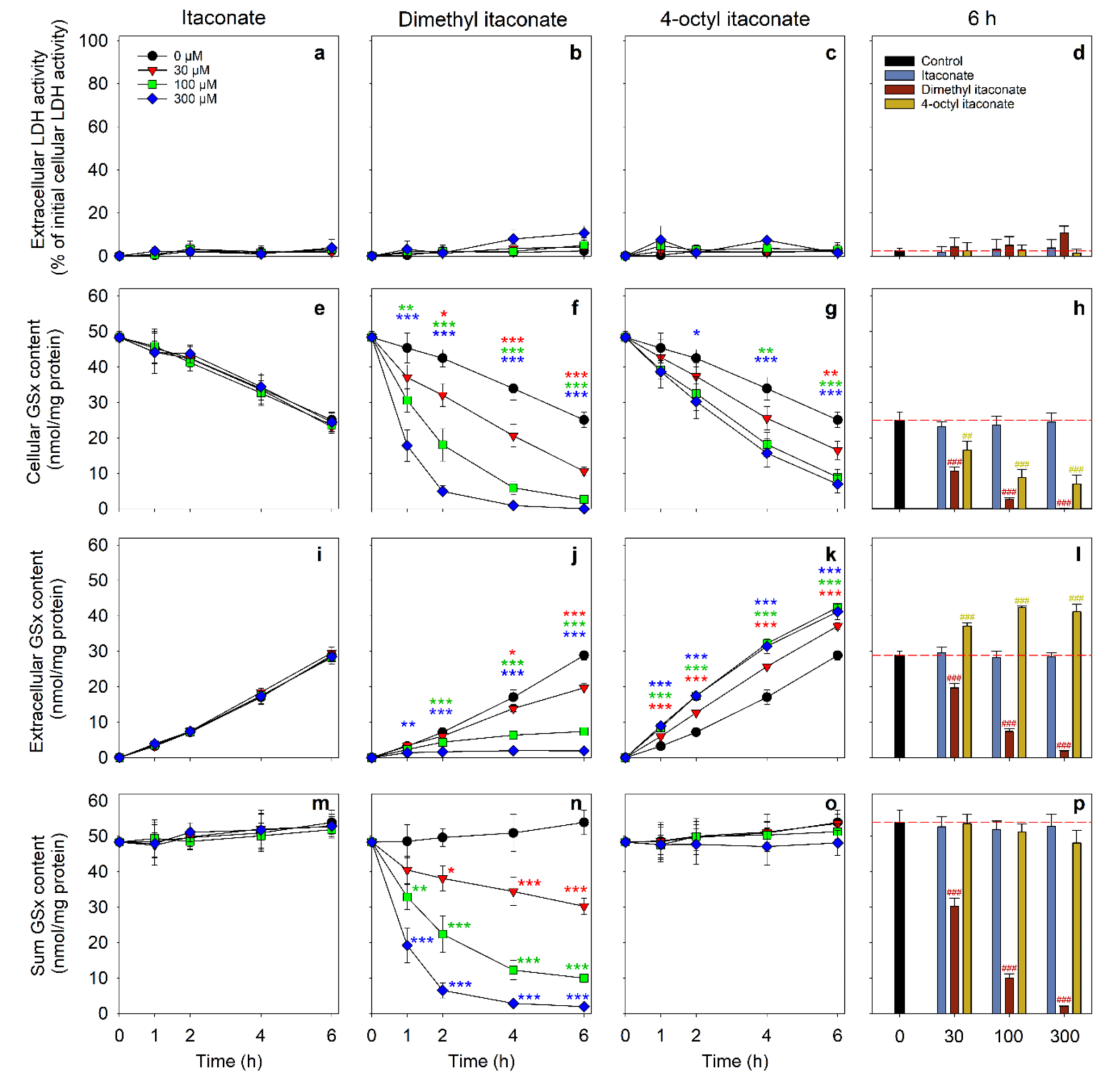
Test for the consequences of an exposure with itaconate and its derivatives on the viability and the GSx content of cultured astrocytes. The cells were incubated without (0 µM) or with the given concentrations of itaconate, dimethyl itaconate or 4-octyl itaconate in a glucose-containing (5 mM) incubation buffer with 100 µM acivicin for up to 6 h. The extracellular LDH activity (**a**-**d**), the cellular (**e**-**h**) and extracellular (**i**-**l**) GSx contents and the sum of cellular plus extracellular GSx contents (**m**-**p**) were determined for the indicated incubation times. The initial specific cellular GSx content was 48.4 ± 1.6 nmol/mg protein and the initial protein content of the cultures was 149 ± 8 µg/well. The data presented are means ± SD of values from experiments that had been performed on three independently prepared astrocyte cultures. The significance of differences (ANOVA) compared to the control incubation (absence of itaconate and its derivatives) is indicated by *p < 0.05, **p < 0.01 and ***p < 0.001 in the colours of the respective symbols for the given concentrations. In the concentration dependencies shown for the three itaconates (d, h, l and p), the significance of differences (ANOVA) of values compared to the respective concentration of itaconate was calculated and is indicated by ^##^*p* < 0.01 and ^###^*p* < 0.001 in the colours of the symbols used for the itaconate derivatives

To test whether an application of itaconate, DI or OI may affect cell viability and the cellular GSH metabolism, astrocyte primary cultures were incubated in the absence or the presence of itaconate, DI or OI in concentrations of 30 µM, 100 µM and 300 µM for up to 6 h. None of these incubations compromised the cell viability as demonstrated by the absence of any increase in extracellular LDH activity (Fig. 1a-d). During the 6 h incubation, astrocytes that were incubated without itaconate, DI or OI exported about half of their GSH as shown by the decrease in the cellular GSx content from initially 48.4 ± 1.6 nmol/mg protein to 25.1 ± 2.2 nmol/mg protein (Fig. 1e) and by the appearance of 28.8 ± 1.3 nmol GSx/mg protein in the incubation medium (Fig. 1i), while the sum of cellular plus extracellular GSx contents remained unaltered throughout the incubation period (Fig. 1m). The respective data for incubations of astrocytes with itaconate in concentrations of up to 300 µM were almost identical to those found in the absence of itaconate (Fig. 1e, i, m). In contrast, the presence of DI resulted in a time- and concentration-dependent loss in the cellular and extracellular GSx contents (Fig. 1f, j), thereby lowering the total amount (cellular plus extracellular values) of GSx in the cultures (Fig. 1n). Already after 4 h of incubation with 300 µM DI, the cells were almost completely deprived of GSx (Fig. 1f), and hardly any extracellular GSx was detectable for this condition (Fig. 1j). The exposure of astrocytes with OI resulted also in an accelerated loss of cellular GSx (Fig. 1g), but the determined decline in cellular GSx was accompanied by a matching increase in the extracellular GSx content (Fig. 1k) and the sum of cellular plus extracellular GSx contents remained unaltered throughout the entire incubation period for each of the applied concentrations of OI (Fig. 1o).

Direct comparison of the data obtained for the 6 h incubations with itaconate, DI or OI revealed that itaconate in concentrations of up to 300 µM did not affect cellular (Fig. 1h) and extracellular (Fig. 1l) GSx contents, while both DI and OI accelerated cellular GSx loss in a concentration-dependent manner (Fig. 1h). However, both itaconate derivatives differed strongly in their effect on the extracellular accumulation of GSx, as OI accelerated extracellular GSx accumulation, while DI lowered this process in a concentration-dependent manner (Fig. 1l).

GSx represents the sum of GSH plus twice the amount of GSSG [38]. Analysis of the contribution of GSSG to the determined GSx contents for cells that had been treated without itaconate and its derivatives revealed that after a 6 h incubation, GSSG represented only around 3% of the determined cellular GSx and around 10% of the extracellular GSx (Table 1), demonstrating that GSH accounts for the large majority of intra and extracellular GSx contents and that the cells were not under any oxidative stress, consistent with literature data [34, 45]. An exposure of astrocytes with itaconate or its derivatives DI or OI did also not cause any significant increase in specific GSSG values (Table 1). The apparent increase in the percental extracellular GSSG content of cells that had been treated with DI (Table 1) is likely to be the consequence of the very low contents of GSx and GSSG determined for this condition which are in the range of the detection limit of the assay used.

**Table 1:** Test for the consequences of an exposure of cultured astrocytes with itaconate or its derivatives on the cellular and extracellular contents of GSx and GSSG

Substance	Cellular contents			Extracellular contents		
	GSx	GSSG	GSSG	GSx	GSSG	GSSG
	(nmol/mg)	(nmol/mg)	(% of GSx)	(nmol/mg)	(nmol/mg)	(% of GSx)
Control	25.1 ± 2.2	0.8 ± 0.3	13 ± 1	28.8 ± 1.3	2.6 ± 0.3	9 ± 1
Itaconate	24.4 ± 2.6	0.7 ± 0.5	13 ± 2	28.4 ± 1.1	2.4 ± 0.1	8 ± 1
Dimethyl itaconate	0.0 ± 0.0***	0.6 ± 0.2	n. c.	1.9 ± 0.2***	0.5 ± 0.1***	25 ± 9**
4-octyl itaconate	6.9 ± 2.5***	0.6 ± 0.2	10 ± 5	41.2 ± 2.2***	3.2 ± 0.4	8 ± 1

The cells were incubated without (0 µM) or with 300 µM itaconate, dimethyl itaconate or 4-octyl itaconate in a glucose-containing (5 mM) incubation buffer with 100 µM acivicin for 6 h before the cellular and extracellular contents of GSx and GSSG were determined. The initial specific GSx content was 48.4 ± 1.6 nmol/mg protein and the initial protein content of the cultures was 149 ± 8 µg/well. The data presented are means ± SD of values from experiments that had been performed on three independently prepared astrocyte cultures. The GSx contents are those already shown in Fig. 1. The significance of differences (ANOVA) compared to the control incubation (none) is indicated by *p < 0.05, **p < 0.01 and ***p < 0.001. n.c. = not calculable

### Effects of the Mrp1-inhibitor MK571 on the GSx Export for OI-treated Astrocytes

**Fig. 2:**
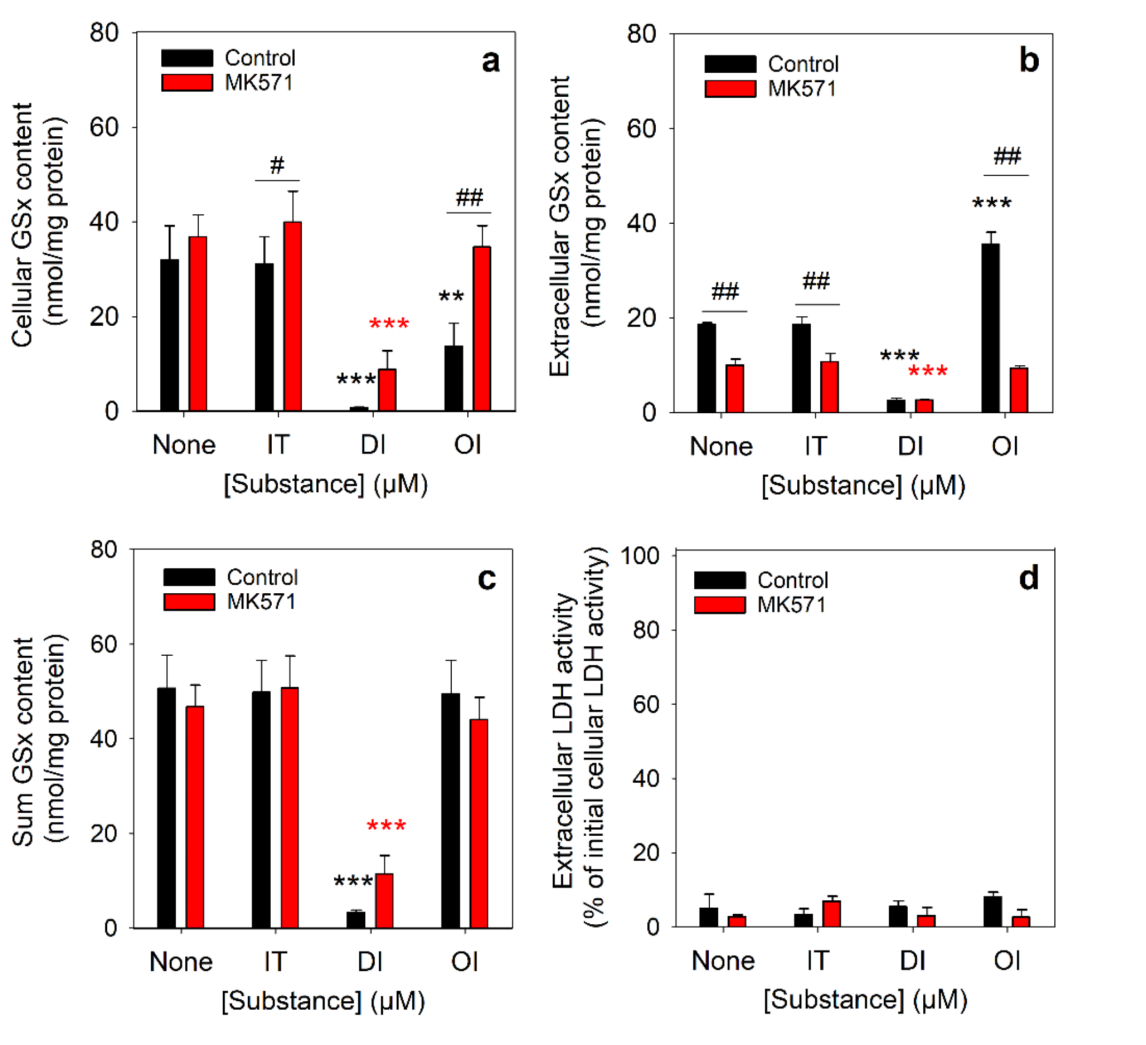
Test for the consequences of a MK571-mediated Mrp1 inhibition on the cellular and extracellular GSx contents of itaconate-treated astrocytes. The cells were incubated in a glucose-containing (5 mM) incubation buffer for 4 h without (none) or with 300 µM of itaconate (IT), dimethyl itaconate (DI) or 4-octyl itaconate (OI) with 100 µM acivicin in the absence (control) or the presence of 50 µM of the Mrp1 inhibitor MK571. The cellular (**a**) and extracellular (**b**) contents of GSx, the sum of cellular plus extracellular GSx contents (**c**), and the extracellular LDH activity (**d**) were determined. The initial specific GSx content was 49.5 ± 5.9 nmol/mg protein and the initial protein content of the cultures was 150 ± 17 µg per well. The data presented are means ± SD of values from experiments that had been performed on three independently prepared astrocyte cultures. The significance of differences (ANOVA) compared to the respective control incubation (none) was analysed and is indicated by **p < 0.01 and ***p < 0.001. The significance of differences (t-test) of data obtained for incubations in the absence or the presence of MK571 is indicated by ^#^*p* < 0.05 and ^##^*p* < 0.01

GSH export from astrocytes is mainly mediated by Mrp1 [30, 33]. To test whether Mrp1 is involved in the stimulated GSH export observed for OI-treated astrocytes, the cells were incubated for 4 h without or with itaconate or its derivatives in the absence or the presence of the Mrp1 inhibitor MK571. In the absence of itaconate, DI and OI, the cells exported within 4 h about 38% of their initial cellular GSx content resulting in cellular and extracellular GSx values of 32.0 ± 7.5 nmol/mg protein and 18.7 ± 0.3 nmol/mg protein, respectively (Fig. 2a, b). In contrast, after incubation with 50 µM MK571 for 4 h, the extracellular GSx content was with 10.0 ± 1.3 nmol/mg protein (20% of the initial cellular GSx content) half of that found for the respective incubation without MK571 (Fig. 2b). Incubation with MK571 did not alter the sum of cellular plus extracellular GSx levels compared to the control condition (absence of MK571) which remained similar to the initial cellular GSx content (Fig. 2c). Almost identical results to that of the incubation of control cells (absence of itaconate, DI and OI) were found for incubations of astrocytes with 300 µM itaconate in the absence or the presence of MK571 (Fig. 2a-c).

After a 4 h incubation of astrocytes with 300 µM DI, neglectably small amounts of cellular and extracellular GSx levels were detected (Fig. 2a-c). The presence of MK571 appears to prevent part of the cellular GSx loss, but the observed difference did not reach the level of significance (Fig. 2a). In contrast, the accelerated loss in cellular GSx of OI-treated astrocytes and the stimulated extracellular accumulation of GSx were completely prevented by the presence of MK571 (Fig. 2a, b), resulting in total (cellular plus extracellular) GSx contents that were nearly identical to the contents of control cells that had been incubated in the absence of OI (Fig. 2c). None of the conditions used compromised cell viability as demonstrated by the absence of any increase in extracellular LDH activity (Fig. 2d).

### Test for Cell-independent Chemical Reaction of GSH with Itaconate and its Derivatives

**Fig. 3:**
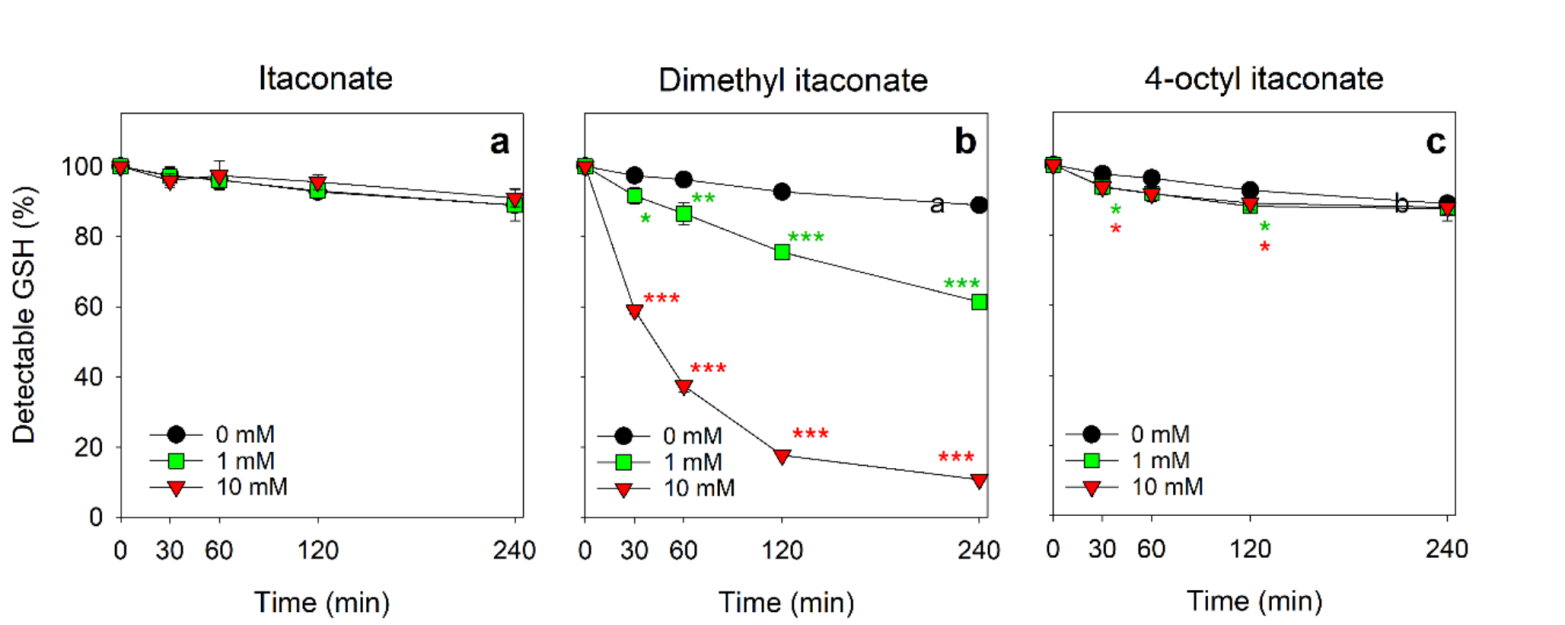
Test for cell-independent reactions between GSH and itaconate, dimethyl itaconate or octyl itaconate. GSH (10 µM) was incubated without (0 mM) or with 1 mM or 10 mM itaconate, DI or OI in 1 mL IB for up to 240 min. The GSH content was determined for the indicated time points. The data presented are means ± SD of values from three independent experiments. The significance of differences (ANOVA) compared to the respective control incubation (absence of itaconate and its derivatives) was analysed and is indicated by *p < 0.05, **p < 0.01 and ***p < 0.001

In order to investigate whether GSH chemically reacts in the absence of cells with itaconate or its derivatives under the conditions used for cell experiments, 10 µM GSH were incubated with a large excess of itaconate, DI or OI for up to 4 h. Excess of itaconate or OI did hardly affect the detectable amount of GSH during the incubation (Fig. 3a, c), while the presence of DI lowered in a time-dependent manner the detectable amount of GSH within 4 h by 31% (1 mM DI) and by 88% (10 mM DI) (Fig. 3b).

### Investigation of a Potential Interference of Itaconates with the Glycolytic Lactate Production in Astrocytes

**Fig. 4:**
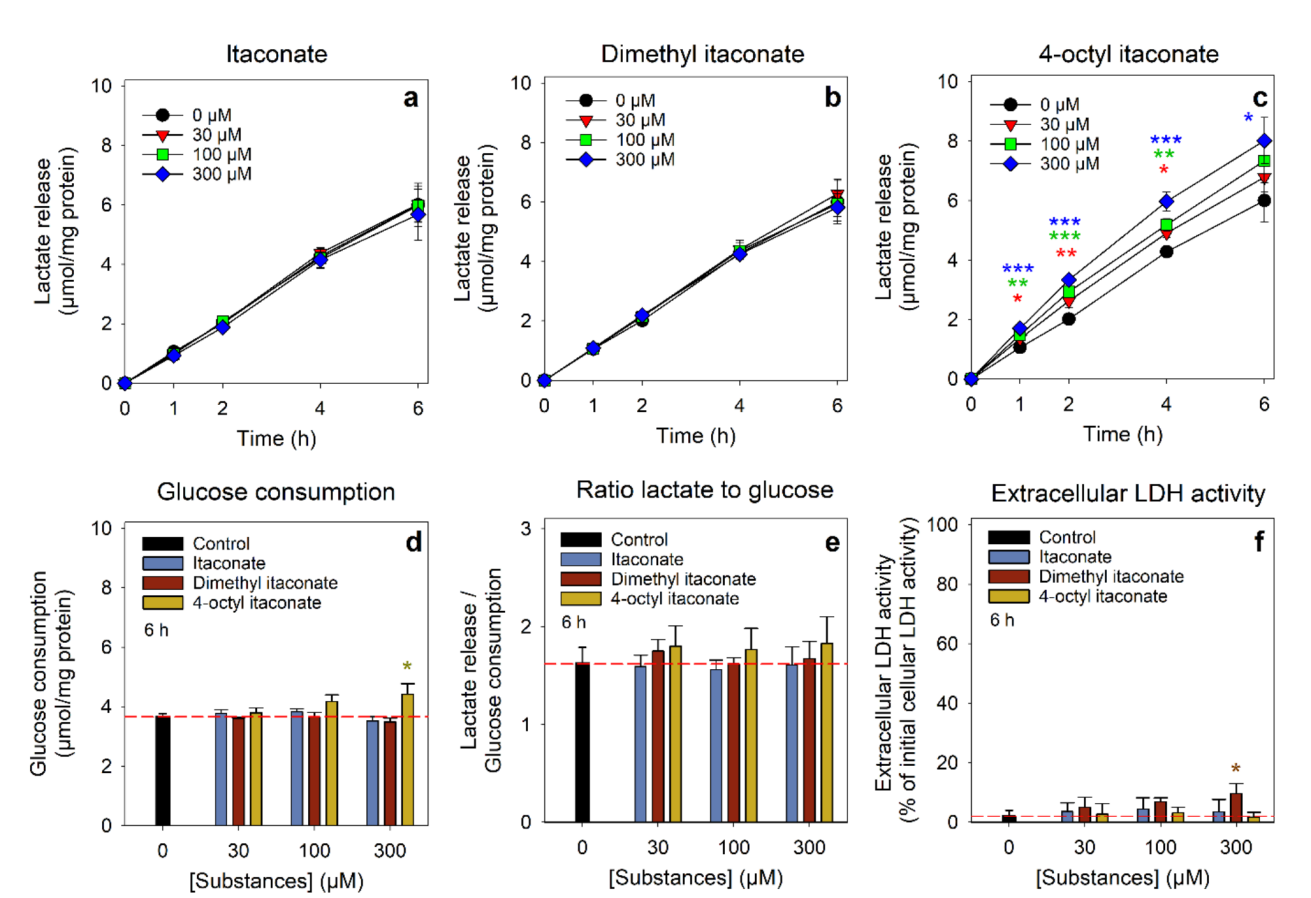
Test for potential effects of itaconate and its derivatives on the glycolytic lactate generation by cultured astrocytes. The cells were incubated without (0 µM) or with the given concentrations of itaconate (**a**, **d**-**f**), dimethyl itaconate (**b**, **d**-**f**) or octyl itaconate (**c**, **d**-**f**) in a glucose-containing (5 mM) incubation buffer with 100 µM acivicin for up to 6 h. The specific extracellular lactate content (**a**-**c**) and the extracellular LDH activity for the 6 h incubation period (**f**) was determined for the indicated time points. In addition, the specific glucose consumption (**d**) and the ratio of lactate release to glucose consumption (**e**) were calculated for the 6 h values. The initial protein content of the cultures was 136 ± 22 µg/well. The data shown represent means ± SD of values from experiments that had been performed on three independently prepared astrocyte cultures. The significance of differences (ANOVA) compared to the control incubation (0 µM) is indicated by *p < 0.05, **p < 0.01 and ***p < 0.001 in the colours of the symbols used for the respective concentrations or compounds

To test for a potential effect on the glycolytic lactate production, cultured astrocytes were exposed to 5 mM glucose without or with itaconate, DI or OI in concentrations of up to 300 µM for up to 6 h. During incubation of control cells (absence of itaconate and its derivatives), lactate accumulated almost proportional to time for up to 6 h in the incubation medium (Fig. 4). After 6 h, the cells had released 6.0 ± 0.7 µmol lactate/mg protein (Fig. 4a-c) and had consumed 3.7 ± 0.1 µmol glucose/mg protein (Fig. 4d). The presence of itaconate or DI in concentrations of up to 300 µM during the incubation did not alter lactate release (Fig. 4a, b) nor glucose consumption (Fig. 4d), compared to the results obtained for control cells. In contrast, incubation of astrocyte primary cultures with OI resulted in significantly elevated extracellular lactate contents already after 1 h of incubation with 30 µM OI (Fig. 4c). After 6 h of incubation with 300 µM OI, the cellular lactate release was found increased by 33% to values of 8.0 ± 0.8 µmol lactate/mg protein (Fig. 4c), and the specific glucose consumption was significantly increased by around 20% compared to control conditions (Fig. 4d). The ratio of lactate released to glucose consumed remained unaltered for all tested substances and conditions (Fig. 4e). None of the conditions applied caused any substantial increase in the extracellular LDH activity (Fig. 4f), demonstrating that the cell viability was not compromised.

### Test for Potential Interference of Itaconates with the ß-lapachone-mediated WST1 Reduction

**Fig. 5:**
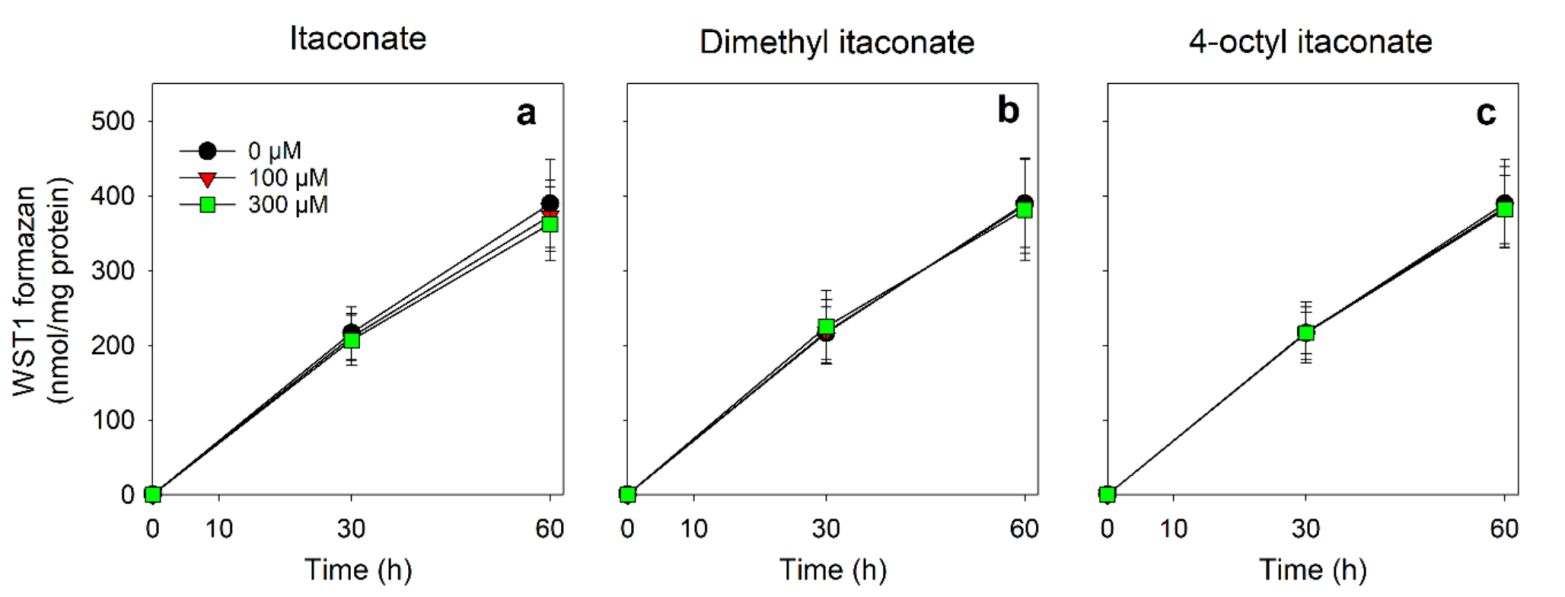
Test for potential effects of itaconate and its derivatives to affect the β-lapachone-mediated WST1 reduction of cultured astrocytes. The cells were pre-incubated in a glucose-free incubation buffer for 30 min and subsequently incubated without (0 µM) or with 100 µM or 300 µM of itaconate (**a**), dimethyl itaconate (**b**) or 4-octyl itaconate (**c**) in a glucose-containing (5 mM) incubation buffer containing 3 µM β-lapachone and 400 µM WST1 for up to 60 min. The extracellular WST1 formazan content was determined after the indicated time points. The initial protein content of the cultures was 128 ± 18 µg per well. None of the conditions used increased the extracellular LDH activity significantly (Table 2). The data presented are means ± SD of values from experiments that had been performed on three independently prepared astrocyte cultures. The differences of data obtained for the different concentrations and between the tested compounds was not significant (ANOVA)

Astrocytic NQO1-dependent and β-lapachone-mediated WST1 reduction is mainly fuelled by NADPH that is generated from glucose-6-phosphate in the PPP [43, 46]. In order to investigate whether itaconate, DI or OI may affect the astrocytic reduction of WST1 to WST1 formazan, astrocyte cultures were incubated with β-lapachone and WST1 in the absence or the presence of 100 µM or 300 µM of itaconate, DI or OI for up to 60 min. In the absence (0 µM) of itaconate, DI and OI a time-dependent linear increase in extracellular WST1 formazan was observed that reached after 60 min nearly 400 nmol/mg protein (Fig. 5a-c). This WST1 reduction by astrocytes was not affected by the presence of 100 µM or 300 µM itaconate, DI or OI (Fig. 5a-c; Table 2). In contrast, the presence of the glucose-6-phosphate dehydrogenase (G6PDH) inhibitor G6PDi-1 [46] significantly lowered the cellular WST1 reduction by around 55% (Table 2). Co-application of 300 µM of itaconate, DI or OI with G6PDi-1 did neither alter the inhibitory potential of G6PD-1 on WST1 reduction nor the rate of G6PDH-independent (data determined in the presence of G6PDi-1; [46]) electron supply for WST1 reduction (Table 2).

**Table 2:** Test for potential effects of itaconate and its derivatives on the G6PDH-independent β-lapachone-mediated WST1 reduction by cultured astrocytes

	Control		G6PDi-1	
Compound tested	LDH (%)	WST1 formazan (nmol/mg protein)	LDH (%)	WST1 formazan (nmol/mg protein)
None	5 ± 9	390 ± 59	3 ± 4	174 ± 33^##^
Itaconate	1 ± 1	362 ± 49	2 ± 3	160 ± 36^##^
Dimethyl itaconate	1 ± 2	381 ± 67	4 ± 5	175 ± 36^##^
4-Octyl itaconate	5 ± 3	382 ± 46	3 ± 2	168 ± 26^##^

The cells were pre-incubated in a glucose-free incubation buffer for 30 min and subsequently incubated for 60 min without (None) or with 300 µM of itaconate, dimethyl itaconate or 4-octyl itaconate in a glucose-containing (5 mM) incubation buffer (containing 3 µM β-lapachone and 400 µM WST1) in the absence (Control) or the presence (30 µM) of the G6PDH inhibitor G6PDi-1 before the extracellular LDH activity (given as % of the initial cellular LDH activity) and the specific WST1 formazan formation were determined. The data for the WST1 formazan formation in the absence of G6PDi-1 are those already shown in Fig. 5. The initial protein content of the cultures was 128 ± 18 µg per well. The data presented are means ± SD of values from experiments that had been performed on three independently prepared astrocyte cultures. The significance of differences (ANOVA) of data compared to those of the control incubation (None) was tested, but no significant differences were observed. The significance of differences (t-test) of data obtained for incubations in the absence or the presence of G6PDi-1 is indicated by ^##^*p* < 0.01

## Discussion

Primary astrocyte cultures were used to investigate potential adverse consequences of an exposure of astrocytes to itaconate, DI or OI on cell viability, GSH metabolism and glucose metabolism. None of the conditions investigated caused a substantial increase in extracellular LDH activity, demonstrating that incubations of up to 6 h with itaconate, DI or OI in concentrations of up to 300 µM do not compromise cell viability. This confirms the low cell toxic potential of these compounds reported for various cell types [37, 47–51]. In addition, the absence of any detectable increase in cellular GSSG contents in treated astrocytes demonstrates that the presence of itaconate, DI or OI does not cause any detectable oxidative stress under the conditions used. This was expected as itaconate and its derivatives have been reported to rather improve antioxidative defense by activation of Nrf2 in different cell types [52, 53] including astrocytes [36, 37].

Application of itaconate in concentrations of up to 300 µM for up to 6 h did not affect the cellular GSH metabolism, the GSH export, the cytosolic glucose metabolism via glycolysis or PPP. Similarly, after exposure of 250 µM itaconate to unstimulated bone marrow-derived macrophages the cellular GSH content has been reported to remain unaltered and formation of itaconate-GSH conjugates was not found [54]. Due to the low membrane permeability of itaconate [47], the micromolar concentration of extracellular itaconate applied in the present study appears to be insufficient to cause sufficiently high cellular concentrations that are able to affect the metabolic processes investigated.

An increase in endogenous itaconate levels after experimental stroke has very recently been linked to a decrease in pyruvate contents, supporting the hypothesis that itaconate acts as glycolysis inhibitor via competitive inhibition of pyruvate kinase [55]. Considering the low concentrations of itaconate applied in our study (up to 300 µM) and the low membrane permeability of itaconate, the cellular concentration established by the extracellular application of itaconate to cultured astrocytes may be too low to affect glycolysis. Further studies are now required to test for the potential of endogenously produced itaconate to affect the metabolism of astrocytes.

DI and OI have frequently been applied as membrane permeable derivatives of itaconate to study potential effects of itaconate on astrocytes [36, 37] and other cell types [56, 57]. Indeed, application of these compounds affected astrocytic GSH and glucose metabolism. It is assumed that after uptake into cells the ester bonds of DI and OI are hydrolyzed by esterases and itaconate is liberated to affect cell functions [49, 57]. As such hydrolysis of DI or OI will in addition to itaconate also liberate the alcohols methanol or octanol, respectively, a potential contribution of these alcohols to the metabolic effects observed has to be considered. However, as the presence of 300 µM octanol (data not shown) or of up to 1 mM methanol [63] did not affect glycolytic lactate production nor astrocytic GSH metabolism or transport, a potential effect of the alcohols liberated from DI or OI on the parameters investigated in the current study can be excluded.

DI and OI have been reported to affect cytosolic glucose metabolism and mitochondrial energy production in several cell types [35, 58] and the modification of metabolic enzymes by alkylation through the itaconate liberated from the itaconate esters has been discussed to contribute to the reported effects [56]. For cultured astrocytes, the presence of DI did not acutely affect glucose consumption, glycolytic lactate generation nor the supply of electrons by the PPP or other pathways for WST1 reduction, suggesting that for the conditions studied astrocytic enzymes are not rapidly modified by the presence of DI to an extend that would lead to detectable alterations of the processes investigated.

The presence of OI accelerated glycolytic lactate production by cultured astrocytes as reported previously for many drugs that inhibit mitochondrial processes [59, 60], suggesting that also the observed acceleration of lactate production by OI-treated astrocytes may be the consequence of mitochondrial impairment. This would be consistent with literature data reporting that itaconate inhibits succinate dehydrogenase due to its structural similarity to succinate [48, 61, 62]. In astrocytes, the itaconate required for such an inhibition could be generated by hydrolysis from uptaken OI, as reported for other cells [49]. For LPS-stimulated macrophages and colorectal cancer cells was shown that presence of OI rather inhibits glycolysis and that this effect is caused by alkylation of the catalytically active cysteine residue in the active center of the glycolytic enzyme glyceraldehyde 3-phosphate dehydrogenase (GAPDH) [63, 64]. Such an inhibition appears not to take place in OI-treated astrocytes as glycolytic lactate production was not lowered but rather accelerated. However, it should be considered that GAPDH is present in high activity in cultured astrocytes and that the activity of this enzyme has to be inhibited by more than 80% before an impairment of glycolytic lactate production is observed [65].

The normal slow loss of cellular GSH from cultured astrocytes [30, 33] was found accelerated in a time- and concentration-dependent manner by the presence of either DI or OI. However, different mechanisms appear to be responsible for the loss in cellular GSH in presence of the two itaconate esters. Presence of DI caused a decline in GSH contents in both the cells and the medium. As an excess of DI also depleted GSH in a cell-free setting, it is likely that GSH reacts as nucleophile with the α,β-unsaturated carboxylate in a Michael-type addition to form a 2,3-dicarboxypropyl adduct. A similar alkylation of GSH like that found for DI-treated astrocytes has been reported for fumaric acid diesters for cells and cell-free conditions [66, 67]. In contrast to DI, GSH disappearance in cell-free conditions was not found for an excess of OI, consistent with the much lower reactivity of the charged monoesters of itaconate and fumarate with thiols such as GSH [49, 66–68]. Although such Michael-type chemical reaction with GSH can be expected for all α,β-unsaturated carboxylates [56], itaconate and OI have been reported to have far lower reactivity towards thiols than DI [49], explaining why the total GSH content of the treated cultures (cellular plus extracellular content) is only lowered by DI but not by itaconate or OI. Further studies are now required to confirm that indeed a conjugation product between DI and GSH is formed after application of DI to cultured astrocytes and whether this conjugate is further metabolized and/or exported by the cells. It also remains to be elucidated whether in astrocytes the chemical derivatization of GSH in the presence of DI is accelerated by cellular glutathione-S-transferases, which are known to catalyze the reaction of GSH with unsaturated compounds [69, 70].

The accelerated loss in cellular GSH in OI-treated astrocytes was accompanied by a matching increase in the extracellular GSH content, suggesting that the presence of OI stimulates the export of GSH. A similar stimulation of GSH export from cultured astrocytes has been reported for treatments with formaldehyde [71] or antiretroviral protease inhibitors [34, 72, 73] and was shown to be mediated by Mrp1. The application of the Mrp1 inhibitor MK571 [22, 30, 33] completely prevented the OI-stimulated GSH export, demonstrating that also the presence of OI accelerates Mrp1-mediated GSH export from astrocytes. Similar to the endogenous Mrp1 substrate leukotriene C4 [74], OI contains a hydrophobic octyl-group as well as a polar carboxyl-group. Due to the bipartite binding site of Mrp1 [34, 74], such structural features of OI facilitate binding of compounds to Mrp1 which stimulate GSH transport from astrocytes [34].

Itaconate has recently been reported to stimulate the OXGR1 receptor in the respiratory epithelium in a mouse model of pulmonary infection with bacteria [6], demonstrating that itaconate can have extracellular functions as ligand of a receptor. As the presence of OXGR1 mRNA in human brain cells has been reported [75], itaconate might also act as extracellular signaling molecule in brain. This option should be addressed in further studies and a possible differential potential of itaconate, DI or OI to activate OXGR1 and to modify immune responses in brain cells needs to be elucidated [56].

In conclusion, an exposure of cultured astrocytes to itaconate or its membrane-permeable esters DI or OI in concentrations of up to 300 µM did not cause any acute oxidative stress, did not compromise cell viability and did not affect electron supply via the PPP for cell-dependent WST1 reduction. However, the three tested compounds differed strongly concerning their potential to affect glycolytic lactate production, the cellular GSH content and the GSH export. While applied itaconate did not affect the cellular pathways investigated, OI stimulated glycolytic lactate production and Mrp1-mediated GSH export. In contrast, DI depleted the cultures of GSH, most likely by chemical addition of the thiol to the double bond of DI. These strong differences in the consequences of a treatment of astrocytes with itaconate, DI and OI should be considered for future studies that use itaconate esters such as DI and OI as potential extracellular sources to increase cellular itaconate levels as well as for the design of strategies to use itaconate esters as therapeutic agents to limit detrimental effects after ischemic brain injury or stroke.

## Data Availability

No datasets were generated or analysed during the current study.
